# Climate change, food insecurity, and the impacts on child health and nutrition in Brazil: proposal for a conceptual model

**DOI:** 10.1590/0102-311XEN217824

**Published:** 2025-12-01

**Authors:** Lissandra Amorim Santos-Degner, Poliana de Araújo Palmeira, Elisabetta Gioconda Iole Giovanna Recine, Elaine Martins Pasquim, Rosana Salles-Costa, Ana Maria Segall-Corrêa, Janaína Braga de Paiva, Larissa Ferreira Tavares Nonato, Sandra Maria Chaves dos Santos

**Affiliations:** 1 Universidade Federal da Bahia, Salvador, Brasil.; 2 Universidade Federal de Campina Grande, Campina Grande, Brasil.; 3 Universidade de Brasília, Brasília, Brasil.; 4 Ministério da Ciência, Tecnologia, Inovação e Comunicações, Brasília, Brasil.; 5 Instituto de Nutrição Josué de Castro, Universidade Federal do Rio de Janeiro, Rio de Janeiro, Brasil.; 6 Faculdade de Ciências Médicas, Universidade Estadual de Campinas, Campinas, Brasil.; 7 Centro de Ciências da Saúde, Universidade Federal da Paraíba, João Pessoa, Brasil.

**Keywords:** Climate Change, Child Malnutrition, Food Insecurity, Food System, Syndemic, Cambio Climático, Desnutrición Infantil, Inseguridad Alimentaria, Sistema Alimentario, Sindémico

## Abstract

Climate change has led to an increase in the frequency and intensity of extreme weather events, forcing approximately 72 million people throughout the world to face limitations in terms of access to food in 2023. In Brazil, this scenario intensifies situations of chronic hunger, poverty, and social inequalities, increasing vulnerability particularly among young children. The aim of this paper is to propose a conceptual model that elucidates and explains these relationships. A literature review was conducted using the terms “climate change”, “food insecurity”, “child malnutrition”, and combinations of these terms, with the inclusion of the word “Brazil” in order to discuss the model in the Brazilian context. A conceptual model was proposed that addresses relationships among the main elements and three other mediating factors - the food system, water insecurity, and social inequalities, which are interrelated in five ways: (a) the direct impact of extreme weather events on access to food; (b) the impact of extreme weather events on access to food due to the effects on the food system; (c) water insecurity as an element that adds complexity to the relationship between extreme weather events and food insecurity; (d) social inequalities as determinants of the effect of climate change on households in situations of food insecurity, little access to water, and/or child malnutrition; and (e) child health and nutrition affected by all these factors. The connections addressed in this model can help guide future studies, favoring the development and implementation of collaborative, multisectoral, adaptational actions for the strengthening of resilience to climate change in Brazil.

## Abstract (es)

El cambio climático viene incrementando cada vez más los fenómenos meteorológicos extremos, como los que causaron limitaciones a nivel mundial en el acceso de alimentos a aproximadamente 72 millones de personas en 2023. Este escenario en Brasil agrava las situaciones de hambre crónica, pobreza y desigualdad social, especialmente al aumentar la vulnerabilidad de los niños más pequeños. Este estudio tiene como objetivo proponer un modelo conceptual que exponga y explique estas relaciones. Se realizó una búsqueda bibliográfica con los términos “cambio climático”, “inseguridad alimentaria” y “malnutrición infantil”, y la combinación entre ellos con la inclusión de la palabra “Brasil”, con el fin de discutir el modelo en el contexto de la realidad brasileña. Se propuso un modelo conceptual que presenta las relaciones entre los elementos principales y otros tres elementos de mediación y/o complejidad -sistema alimentario, inseguridad hídrica y desigualdades sociales- que se relacionan de cinco maneras: (a) impacto directo de los fenómenos meteorológicos extremos en el acceso a los alimentos; (b) impacto de los fenómenos meteorológicos extremos en el acceso a los alimentos, debido a sus efectos en el sistema alimentario; (c) inseguridad hídrica como un elemento que agrega complejidad a la relación de los extremos con la inseguridad alimentaria; (d) desigualdades sociales como determinantes del efecto del cambio climático en los hogares en condiciones de inseguridad alimentaria, bajo acceso al agua y/o malnutrición infantil; (e) salud y nutrición infantil afectadas por todos estos factores. Las conexiones discutidas en el modelo pueden guiar otros estudios y posibilitar el desarrollo e implementación de acciones colaborativas y multisectoriales para adaptarse y construir resiliencia al cambio climático en Brasil.

## Introduction

The rise in global temperature is a reality and each year approaches the critical 1.5ºC above the levels of the pre-industrial period (1850-1900) outlined in the Paris Agreement and reports from the Intergovernmental Panel on Climate Change (IPCC). Together with the greater frequency and intensity of extreme weather events, global warming composes what is denominated climate change ^1^. This set of climate changes has a dynamic and complex nature that points to a rapid rise with catastrophic impact on biomes and human food and health, therefore being considered a pandemic ^2^.

One of the most significant impacts of climate change is on food production, which, combined with water scarcity, the socioeconomic vulnerability and low resilience of some populations, and the insufficiency of public policies directed at adaptation and mitigation, has exacerbated hunger throughout the world ^3^ ^,^ ^4^ ^,^ ^5^ ^,^ ^6^ ^,^ ^7^ ^,^ ^8^. On the global scale, climate change exposed approximately 72 million people to food insecurity in 2023 due to limitations in access to food ^9^. Consequently, the prevalence of all forms of malnutrition - including undernutrition and overweight/obesity - has been increasing throughout the world.

The interaction among the pandemics of climate change, undernutrition, and obesity, is conceptualized as a global syndemic, simultaneously affecting a significant share of the world’s population. The health of children under five years of age is particularly at risk due to their physiological immaturity and high nutrient needs ^2^. Globally, approximately 148.1 million children in this age group (22.3%) had stunted growth in 2022, while low weight for height affected 5 million (6.8%) and excess weight affected 37 million (5.6%) ^10^.

In Brazil, child malnutrition rates reflect the higher prevalence and severity of food insecurity found in households with the presence of children ^11^ ^,^ ^12^. In 2019, stunted growth affected 7% of Brazilian children less than five years of age, low weight for age affected 2.9%, and excess weight affected 10.1% ^13^. The coexistence of hunger, as a political and social issue, alongside undernutritions and obesity highlights the concerning nature of the syndemic in Brazil. This context is compounded by structural social inequalities in the country and determines the distribution and severity of food insecurity in Brazilian households ^11^ ^,^ ^12^ ^,^ ^14^ ^,^ ^15^ ^,^ ^16^.

Hence, the challenge lies in elucidating the relationships that may be established between extreme weather events and the impacts on the human right to adequate food as well as child health and nutrition. Some studies have addressed the effects of climate change on human health and nutrition ^17^ ^,^ ^18^. However, gaps remain in the theoretical-scientific investigation of connections between these aspects and both social inequalities and the experience of hunger and food insecurity.

Considering the historical context of social inequalities and the possibility of aggravating child malnutrition, the aim of this paper is to propose a conceptual model that contributes to the discussion on relationships between climate change (with its extreme weather events) and food insecurity as well as the health and nutritional status of Brazilian children.

## Methods

This paper is part of a larger study entitled *Indicators of Climate Change, Food Insecurity, and Hunger in Brazil and Impacts on Child Health and Nutrition* which unites researchers from different institutions with the purpose of developing the FomeS platform (https://fomes.net.br/), composed of indicators created from public national databases. The platform will be destined for use by the scientific-institutional community and interested social movements, fostering the filling of scientific gaps in the dimensions addressed.

The conceptual model characterizes the first step of the study and proposes to establish its initial references from a literature review to describe existing knowledge and identify gaps related to the topic ^19^. To reconstruct the complex relationships between the dimensions of climate change, food insecurity, and child malnutrition on a conceptual level, the model constitutes the starting point for the identification of other related dimensions and the selection of the indicators that will compose the FomeS platform.

### Search strategy

The first step consisted of a search of previously selected databases (PubMed, Web of Science, and SciELO) from February to August 2024, using index terms in English, Spanish, and Portuguese. The search strategy was the combination of three large blocks of investigation terms: child health and nutrition, food security/food insecurity, and climate change or extreme weather events (such as extreme heat waves, droughts, forest fires, and floods). The word “Brazil” was combined with each of these blocks to discuss the proposed model within the Brazilian context. Reports, bulletins, and technical studies were selected, along with peer-reviewed articles from different countries, totaling 150 documents whose most relevant content was used for the creation of the model.

### Systematization and creation of the conceptual model

For systematization, the definition of “food security” was based on various conceptual perspectives, considering agricultural volume and production as well as food availability, access, consumption, and the biological utilization of food by individuals. The studies retrieved from the search of the databases addressed the impact of climate change on food and nutrition security and were conducted with small groups of individuals or data from national surveys - conducted mainly in Asian and African countries - from the perspective of food production or consumption. As no empirical investigations were found in Brazil, two studies offering projections with regards to the possible impact of climate change on national food production were systematized for discussion (Box 1).

**Box 1:** Studies on the relationship between climate change and food security/insecurity according to country.

COUNTRY	EXTREME WEATHER EVENT	DIMENSIONS OF FOOD SECURITY	METHOD/ANALYSIS CATEGORIES	STUDY (YEAR)
Argentina, Kazakhstan, Marocco and South Africa	Variations in temperature, rainfall intensity, and drought	Food production	Comparative analysis of observed grain production records with estimated counterfactual and factual distributions for agricultural productivity according to region and occurrence of extreme and long-term weather events	Food and Agriculture Organization ^4^ (2023)
Brazil	Projected climate change	Food production	Impact analysis of projected climate change on pollinators of 13 crops using computational methods to determine potential areas of occurrence of species and predict their future distribution	Giannini et al. ^84^ (2017)
Brazil	Projected temperature and precipitation data according to scenarios proposed by IPCC (RCP 4.5 and RCP 8.5)	Food production	Cross-section estimation of a production function in which the results in terms of agricultural productivity are determined by climatic, geographic and productive factors	Tnure et al. ^58^ (2024)
China	Average daily temperature and variations in temperature, precipitation, average wind speed, relative humidity, and evaporation	Food production Food availability and consumption	National food security measured in five groups of indicators: quantitative security (grain production); qualitative security (fertilizer and pesticide use); ecological environmental security (grain disaster rate); economic security (price); and resource security (planting area and water for grain production)	Zhou et al. ^26^ (2024)
Mexico	Rains and storms	Food production	Assessment of variability in precipitation and corn yields in Mexico in three different time periods: present, last 30 years, and future RCP scenarios	Murray-Tortarolo et al. ^29^ (2018)
Nepal and Uganda	Monthly variations in precipitation	Biological utilization of food	Assessment of linear growth and weight gain of children under 5 years of age with variation in rainfall during key periods of the phase	Shively ^34^ (2017)
Peru (Iquitos, Loreto)	El Niño Southern Oscillation (ENOS)	Food consumption	Longitudinal observational cohort study (2010-2014) with children nine to 36 months of age and application of 24-hour recall	Ambikapathi et al. ^33^ (2021)
Uganda	Prolonged extreme drought, heat waves, irregular seasons	Food production Food availability and consumption	Qualitative study with indigenous and non-indigenous women from rural areas of Uganda	Bryson et al. ^66^ (2021)
East Timor, Nepal, Philippines, Nigeria, Liberia, Ghana, Madagascar, Uganda, Zambia, Zimbabwe, Namibia, Swaziland, Lesotho, Egypt, Peru, Guyana, Colombia, Dominican Republic and Haiti	Variations in temperature and precipitation	Food availability and consumption	Data from studies in 19 countries - child dietary diversity 30 years of climate data	Niles et al. ^85^ (2021)

IPCC: Intergovernmental Panel on Climate Change; RCP: representative concentration pathways.

The Brazilian concept of food and nutrition security was coined in 2006 ^20^ and used as a guide for the discussion. The terms “food security” and “food insecurity” were used in reference to the classification of families in terms of access to or the deprivation of food, resulting from the application of the *Brazilian Food Insecurity Scale* (EBIA, acronym in Portuguese), which is a measure of hunger and food insecurity in the country ^21^ ^,^ ^22^. Based on the EBIA, families with regular, permanent access to food of sufficient quality and quantity are considered to be in a situation of food security. In contrast, inadequate access to food indicates of food insecurity, which is classified on three levels: mild, moderate, or severe, with severe food insecurity indicative of situations in which hunger is experienced in the household, including by children ^22^.

### Internal validation of the conceptual model content

To achieve consensus on theoretical parameters in the development of the proposed conceptual model, a virtual workshop was held with the team members. The workshop consisted of three steps: (i) sending a link to the theoretical framework for reading and comments prior to the meeting; (ii) systematized presentation of the theoretical framework and figure of the conceptual model; and (iii) dialogue among the participating researchers for adjustments to the model. Eleven members participated in the activity: three researchers from the field of Data Science and eight from the fields of Health and Food and Nutrition Security. The workshop ensured dialogue among the team members, culminating in the validation of both a glossary and a proposed conceptual model, which considered the dimensions identified and their definitions, as well as interrelationships among the elements.

## Results

The terms that guided the creation of the conceptual model are listed in the glossary (Box 2). The model is based on the complex-systems approach, which recognizes interconnections among the elements of a system, by which a change to one of the elements exerts an impact on internal and external environments. Thus, rather than analyzing isolated parts, this approach seeks to understand the whole, considering the resulting relationships, interactions, and emergencies ^17^ ^,^ ^23^.

**Box 2:** Glossary of terms used in conceptual model of interrelations among extreme weather events, food insecurity, and childhood malnutrition.

TERMS	DEFINITION		
Global warming	“*Process of the increase in the temperature near the surface of the planet caused by the presence of greenhouse gases*” ^86^ (p. 3)		
Biome	“*Groups of ecosystems with neighboring vegetation types that exhibit similar geographic and climatic aspects. Brazil has six biomes: Amazon, Caatinga (dry forest in semiarid region), Cerrado (savanna), Atlantic Forest, Pampas (grasslands), and Pantanal (wetlands). The Coastal-Marine System, encompassing the coastline and territorial waters, is considered the 7th Brazilian biome*” ^86^ (p. 4)		
Climate	“*Generally defined as the long-term regional or even global average (typically the average is 30 years) temperature, humidity as well as precipitation and wind patterns over a period of time ranging from months or seasons to thousands or millions of years*” ^36^ (p. 544)		
Social inequalities	“*Systematic, persistent differences in access to goods, resources, and opportunities that are established between individuals, social groups, or even entire populations, irrespective of individual talents, capabilities, and efforts. Social inequalities can be class-based, ethno-racial, or gender-based*” ^87^ (p. 298)		
Deforestation	“*Conversion of native vegetation for pastures and agricultural crops*” ^88^ (p. 46)		
Greenhouse gases	“*Gas components of the atmosphere (H*_*2*_ *O, CO*_*2*_ *, N*_*2*_ *O, CH*_*4*_ *, O*_*3*_ *, etc.), both natural and anthropogenic, that absorb and emit radiation within the spectrum of thermal infrared radiation emitted by the surface of the Earth, the atmosphere, and clouds*” ^36^ (p. 550-1)		
Greenhouse effect	“*Natural phenomenon that ensures a suitable temperature for life on Earth, caused by the concentration of gases in the atmosphere that form a layer for the passage of solar rays and absorption of heat. The increase in the concentration of these gases thickens the layer, hindering the dispersion of solar radiation and causing greater heat retention*” ^86^ (p. 8)		
El Niño and La Niña	“*Climatic phenomena characterized by warming (El Niño) or cooling (La Niña) of the surface of the Pacific Ocean that cause changes in atmospheric circulation patterns throughout the entire the planet*” ^86^ (p. 8)		
*Brazilian Food Insecurity Scale* (EBIA)	Psychometric scale for the direct measurement of food insecurity in Brazil based on the experiences and perceptions of affected individuals - residents of the household ^22^		
Extreme weather events	“*Occurrence of a climate or weather variable above (or below) a limit value near the upper (or lower) extremities of the range of observed values for the variable (includes extreme heat waves, droughts, forest fires, floods, etc.)*” ^86^ (p. 9)		
Food insecurity	Deprivation of regular, permanent access to quality food in sufficient quantities in families. Based on the perception of the experience of the household in the previous 90 days, the Brazilian Food Insecurity Scale indicates one of the following levels of food insecurity experienced by families: mild, moderate, or severe ^22^		
Household water insecurity	“*Defined as the inability to gain access to and benefit from water of adequate quality and in sufficient quantity for all domestic, human, and production uses, when applicable; i.e., reliable (from the standpoint of the source) and safe (from the standpoint of sanitary quality) water to ensure well-being and a healthy life*” ^89^ (p. 2)		
Childhood malnutrition	Condition that occurs due to an imbalance of nutrients essential for growth and development; when a child’s diet lacks adequate nutrients and energy. Includes all forms of malnutrition (undernutrition evidenced by low weight-for-height, stunted growth, or low weight-for-age; vitamin or mineral deficiencies; over weight, obesity, and diet-related noncommunicable diseases) ^23^		
Climate change	“*Any change in climate over time due to natural variability and/or as a result of human activities. Includes increase in surface temperature of the Earth and changes in precipitation, humidity, and wind, with a greater occurrence of extreme weather events*” ^36^ (p. 544)		
Representative concentration pathways (RCPs)	“*Trajectories of greenhouse gas concentrations used to model the climate. The IPCC* [Intergovernmental Panel on Climate Change] *uses RCPs to create future climate change scenarios*” ^36^ (p. 556)		
Child health and nutrition	Promoting healthy eating habits and prevention of child malnutrition in all its forms, using a systems approach to nutrition. This approach strengthens the capacity of five key systems - food, health, water and sanitation, education, and social protection - to provide diets, services, and practices that support adequate maternal and child nutrition ^23^		
Food security	When all people - at all times - have physical, social, and economic access to sufficient, safe, nutritious food that meets their dietary needs and food preferences for an active, healthy life ^51^		
Food and nutritional security	Assurance of everyone’s right to regular, permanent access to quality food in sufficient quantity without compromising access to other essential needs, based on health-promoting eating practices that respect cultural diversity and are environmentally, culturally, economically, and socially sustainable ^22^		
Water security	Availability of water in sufficient quantity and quality to meet human needs as well as ensure the practice of economic activities and the conservation of aquatic ecosystems, accompanied by an acceptable level of risk related to droughts and floods ^90^		
Global syndemic of obesity, undernutrition, and climate change	“*Synergy among the pandemics of obesity, undernutrition, and climate change that interact with each other, sharing common underlying social factors and producing complex, interrelated consequences, according to the concept proposed by Swinburn and collaborators*” ^2^ (p. 794-5)		
Food system	“*Set of all elements (environment, people, inputs, processes, infrastructure, institutions, etc.) and activities related to the production, processing, distribution, preparation, and consumption of food, and the results of these activities, including socioeconomic and environmental results. The food system includes three subsystems: (i) the food supply chain (encompasses activities such as production, storage, distribution, processing, packaging, retail, and marketing); (ii) the food environment (encompasses dimensions of availability, such as prices, properties of the supplier and product, and personal dimensions, such as accessibility, convenience of food sources and products); and (iii) eating behavior (reflects the choices and decisions made by consumers regarding which foods to select and how to store, prepare, and consume these foods in the home)*” ^91^ (p. 23)		

**Figure 1:**
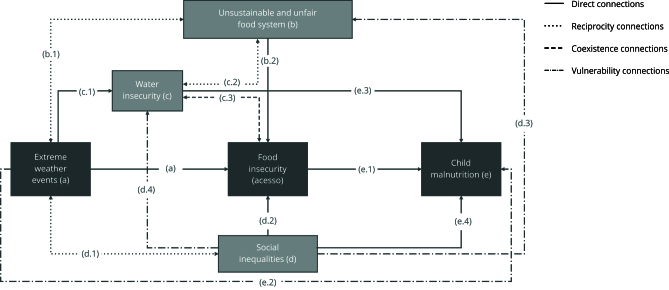
Conceptual model indicating the pathways that climate change - through extreme weather events - takes to impact child health and nutrition, with food insecurity as a mediating factor.

Note: letters (a-e) indicate the different dimensions; numbers (1-4) indicate the different pathways connecting the dimensions. The (a) pathway indicates the direct impact of climate change on food insecurity; the (b) pathways (b.1 and b.2) indicate the relationship between the food system and both climate change and food insecurity; The (c) pathways (c.1 to c.3) indicate the relationship between water insecurity and the other components; the (d) pathways (d.1 to d.4) show how social inequalities determine the relationships; and the (e) pathways (e.1 to e.4) show how malnutrition is influenced by food insecurity, climate change, water insecurity, and social inequalities.

The model identifies pathways in the relationship between climate change (extreme weather events) and child malnutrition mediated by food insecurity status and other factors, such as the food system, water insecurity, and social inequalities (Figure 1). The solid lines in the figure correspond to direct connections (pathways a, b.2, c.1, d.2, e.1, e.3, and e.4); dotted lines indicate reciprocal connections (pathways b.1, c.2, and d.1); dashed lines correspond to connections of coexistence (c.3); whereas dashed and dotted lines correspond to connections that increase vulnerability to extreme weather events (d.3, d.4, and e.2).

Taking food insecurity as the central dimension, five other dimensions (a, b, c, d, and e) were defined, along with connecting pathways between dimensions (1 to 4). Pathway (a) considers the emergency impact of the extreme weather events on access to food at a specific point in time, irrespective of the precondition of food insecurity. Pathways b.1 and b.2 consider the mediating role of the food system in the relationship between climate change and food insecurity. This connection has a greater level of complexity when food insecurity is associated with water insecurity (pathways c.1/c.2/c.3). Social inequalities (d) add another layer of complexity by determining how extreme weather events affect the other dimensions (pathways d.1/d.2/d.3/d.4). The last pathways (e.1/e.2/e.3/e.4) regard how children’s health and nutrition are impacted by extreme weather events, food insecurity, water insecurity, and inequalities, generating or aggravating malnutrition among children under five years of age.

## Discussion

### Impact of climate change on food insecurity 

Droughts and floods in the past five years have forced 40% of Brazilian municipalities into states of emergency, which has driven the occurrence of internal migration due to climate-related disasters, resulting in the highest rates in Latin America in 2023 ^24^ ^,^ ^25^. This context aggravates food deprivation, irrespective of prior food insecurity (Figure 1, pathway (a), direct connection).

Studies addressing the impact of climate change on food insecurity are scarce, especially considering hunger experience scales as indicators. A study conducted in China ^26^ revealed that temperature had a linear effect on food security, with higher the temperatures exerting a greater negative impact on this indicator. The study also indicated that precipitation had a non-linear influence, contributing positively to food security indicators when within an optimal range, whereas precipitation above this range would culminate in a negative impact. It should be pointed out that, unlike in Brazil, the concept of food security in China is more closely related to food production indicators ^26^.

In the 1940s, Josué de Castro described the occurrence of epidemic famine in the semiarid region of Northeast Brazil as a consequence of cyclical and extreme droughts in a context of the absence of the government and public policies ^27^. To date, however, no studies have investigated the effect of climate change on food insecurity and hunger in Brazil using the EBIA as one of the indicators. The increased occurrence of extreme weather events calls attention not only to the difficult access to food when there are impacts on production due to the reduction in agricultural crops, but also the immediate impact on income and acute exposure to food insecurity in affected communities, with acute and long-term consequences, especially among the most vulnerable families ^9^ ^,^ ^28^.

### Food systems

Climate change can exert impacts on food security in several ways, especially through the negative effect on food systems ^8^. Factors such as the decline in pollinating insects, the proliferation of pests and pathogens, the greater scarcity of drinking water, and changes in the concentrations of gases and minerals in the atmosphere and soil are consequences of climate change that compromise food production, reducing both the quantity produced and the nutritional content ^5^ ^,^ ^6^ ^,^ ^7^ ^,^ ^8^.

When converted into nutrients, the loss of agricultural products on the global scale due to climatic extremes over the past 30 years is equivalent to the daily dietary needs of approximately 450 million individuals ^4^. Reductions in the micronutrients of foods seem to be particularly significant for iron, phosphorus, magnesium, and thiamine, mainly affecting grains, but also fruit and vegetable crops and sugarcane ^4^ ^,^ ^7^.

There is evidence of a 2% to 25% reduction in the quantity of grains (wheat and corn) produced in countries such as Kazakhstan, Morocco, South Africa, and Mexico due to extreme weather events ^4^ ^,^ ^29^. In Latin America, the greater frequency of heat waves and months of drought in 2021 was associated with an increase of 9.9 million individuals with less access to food ^3^. Fishing and aquaculture industries also recorded impacts, such as a reduction in the size of various fish species, changes in composition ^30^, and the loss of biodiversity ^31^.

In Brazil, the national grain and legume harvest suffered a 7.2% drop in 2024 due to extreme weather events that had occurred the previous year ^32^. Projection studies estimate a 3.1% reduction in average crop productivity for each 1ºC increase in average temperature in the warmest seasons of the year ^33^, in addition to a reduction in the occurrence of pollinators in various Brazilian municipalities ^34^. These predictions apply to diverse food crops, such as corn, beans, coffee, guava, tomatoes, etc. ^33^ ^,^ ^34^.

Nonetheless, the country has been consolidating its position as one of the largest food producers on the world stage. The hegemonic food system in Brazil, however, is characterized by the large-scale, mechanized production of food products - whether commodities or not, with a predominance of livestock farming and monocultures as well as the intensive use of water and pesticides. The globalized distribution of food involves processing, packaging, and transportation that exploit natural resources and emit a greater quantity of greenhouse gases (GHG) ^8^ ^,^ ^28^ ^,^ ^35^. This system favors the production and consumption of ultra-processed foods, stimulates the concentration of land and resources among a small number of individuals/companies, and perpetuates the invisibility of the food culture of traditional populations in Brazil. This scenario contributes to the aggravation of social inequalities ^2^ ^,^ ^35^ and is the main contributor to the total gross GHG emissions in the country (73.7%) ^36^.

Regarding the food environment, inequalities in physical access to adequate healthy foods are notable, especially in the urban peripheries of the country. The uneven distribution of commercial establishments that sell healthy and unhealthy foods contributes to the deepening of social inequalities, as farmers’ markets, local markets, and other places that sell fresh or minimally processed foods at affordable prices are scarcer in more vulnerable regions. Conversely, these areas are dominated by small businesses and retail chains that prioritize the supply of ultra-processed foods, which are often more affordable due to factors such as large-scale production, differentiated tax incentives, and the greater negotiating power of the industry ^37^ ^,^ ^38^ ^,^ ^39^.

This context is characterized as one of the factors that have contributed to the growing consumption of ultra-processed foods observed in the last ten years in Brazil ^40^. Such consumption has seen more significant growth among the lower-income population (3.4 percentage points - p.p.) and Indigenous populations (5.96p.p.) ^40^. Moreover, greater consumption of these foods is inversely associated with adherence to a healthy, sustainable diet ^41^. Hence, the hegemonic food system can be considered unsustainable, unfair, and a promoter of unhealthy eating and lifestyle habits ^2^ ^,^ ^8^ ^,^ ^35^.

Therefore, the relationship between climate change and the food system is bidirectional and mutually determinative. Climate change reduces food production in terms of volume and nutrient quality, and the hegemonic food system - in its current state - contributes to the aggravation of climate change (Figure 1, pathway (b.1), reciprocal connection).

Moreover, the greater frequency and intensity of extreme weather events determine and aggravate the situation of food insecurity from the perspective of access to food in three aspects: (i) instability and reduction in food production, generating lower income for small farmers; (ii) such reduction influences market dynamics, culminating in higher prices for the end consumer, especially for fresh foods; (iii) higher prices for fresh foods favors the consumption and production of ultra-processed foods, making such products even cheaper and more accessible, thus reducing access to quality foods (Figure 1, pathway (b.2), direct connection).

### Water insecurity 

Climate change compromises access to drinking water through several pathways: (i) seawater intrusion into coastal aquifers due to glacial losses and snowmelt; (ii) floods, which contaminate drinking water with pathogens that cause infections; and (iii) frequent, persistent droughts ^1^ ^,^ ^7^ ^,^ ^8^ ^,^ ^42^. The increase in the temperature of the Earth surface also alters the water cycle, culminating in water scarcity, which has been increasing in recent decades ^43^ (Figure 1, pathway (c.1), direct connection).

Deforestation, which is a result of the hegemonic food system, contributes to the acceleration of the water cycle, which results in the aggravation of droughts and flooding ^44^. The means of production, processing, and transportation of food, especially ultra-processed food, involve large volumes of water and the pollution of available water resources ^8^. Moreover, the consumption of ultra-processed foods and meat are associated with a larger water footprint (a 10.1% increase across consumption quintiles) ^43^ ^,^ ^45^ (Figure 1, pathway (c.2), reciprocal connection).

Water scarcity exacerbated by extreme weather events tends to put pressure on the food supply, as approximately 70% of freshwater in the world is used for agriculture ^1^ ^,^ ^46^. The reduction in agricultural productivity due to water shortages affects local food security and, in contexts where agriculture is a subsistence activity, water insecurity can trigger trade-offs between allocating water for agricultural or consumption purposes ^8^ ^,^ ^47^ ^,^ ^48^ and precipitate internal migration movements ^4^ ^,^ ^42^ (Figure 1, pathway (c.2)).

Despite having one of the largest freshwater reserves in the world, Brazil is also at risk of water scarcity due to various factors, irrespective of climate change. The expansion of cropland, which requires intensive irrigation, the spatial variability in water reserves - 70% of the available freshwater in the country is found in the North Region - and the growing demand for water resulting from population growth and industrialization are some of these factors ^49^. Moreover, the country has regions that are highly vulnerable to rising surface temperatures, such as the semiarid region, which is characterized by low average annual rainfall and dry periods lasting up to 11 months. This scenario increases the risk of desertification in the region, ultimately leading to the complete unfeasibility of food crops ^50^.

On the household level, water insecurity compromises the hygiene of the home, individuals, and food, and increases the risk of the transmission of infectious diseases ^51^. National data from the end of 2021 and the beginning of 2022 revealed that approximately 12% of the population had restricted access to water and nearly two-thirds of this portion of the population also experienced quantitative food restriction (moderate to severe food insecurity), demonstrating the association between water and food insecurities in Brazil ^52^. The connection between water insecurity and food insecurity is, therefore, one of coexistence (Figure 1, pathway (c.3)). Both can coexist in the same environment and interact with each other, sharing similar determinants related to socioeconomic and demographic characteristics ^47^.

### Social inequalities 

Along with discriminatory social norms, differences in socioeconomic conditions, housing locations, and access to institutions and services determine the climate vulnerability of specific social groups, which adds to the pre-existing susceptibility to food insecurity and water insecurity and the cycle of injustice perpetuated by the dynamics of the hegemonic food system ^53^.

Climate vulnerability is the propensity or predisposition of an individual or social group to be adversely affected by climate change, resulting from the intersection of three factors: exposure, susceptibility, and adaptive capacity ^53^. In a flood situation, for instance, populations in socioeconomically vulnerable situations have precarious housing located in high-risk areas (greater exposure). These conditions make such individuals more susceptible to the direct impacts of flooding, with a greater likelihood of the loss of assets and housing. Moreover, limited financial resources lower their recovery capacity, thus fueling the cycle of vulnerability ^5^ ^,^ ^53^.

Social inequalities make certain groups more vulnerable to extreme weather events. Moreover, the individuals most affected by the climate crisis are those who contribute least to global warming ^1^. Indeed, the richest 1% of the world population was responsible for 16% of global GHG emissions in 2019, which is equivalent to the emissions of approximately 5 billion people (66% of the global population) ^5^ ^,^ ^53^ ^,^ ^54^ (Figure 1, pathway (d.1), reciprocal connection).

In Brazil, the scenario of social inequalities has historical roots and forms the foundations of the country’s development. The current food production model is - and has always been - based on the unequal distribution of land, food, resources, and opportunities, with production concentrated in a few export-oriented foods and little support for small rural properties ^55^. Consequently, although the country produces enough food to feed approximately 800 million people ^56^, hunger remains a chronic problem concentrated in specific regions and social groups, including rural areas (Figure 1, route (d.2), direct connection).

Households in the North and Northeast of Brazil are the most affected by food insecurity. In 2023, severe food restriction (hunger) affected 7.7% of families located in the North Region, which is nearly four times higher than the rate found in the South Region (2%) ^14^. Additionally, food insecurity is more prevalent in households headed by women ^12^ and by black or brown individuals (69.6% vs. 29%) ^14^, among *Quilombola* communities (86.1% of households) ^16^, and among Indigenous peoples of the State of Mato Grosso do Sul (76.7%) ^15^.

The intersection of these characteristics produces deeper levels of climate vulnerability. Households headed by black women are four times more likely to report moderate or severe food insecurity compared to those headed by white men ^12^. Food insecurity is known to be associated with other indicators of poverty, such as low education, a larger number of residents in the home, and lower income, which further reduces the likelihood of such populations breaking the cycle of poverty and hunger and, therefore, coping with the effects of extreme weather events ^11^ ^,^ ^53^ ^,^ ^57^.

Families living in rural areas are also more vulnerable to food insecurity and extreme weather events. National data from 2023 show that the proportion of severe food insecurity was 1.6p.p. higher in rural areas compared to urban areas (5.5% vs. 3.9%) ^14^. In such areas, family farmers and lower-income households, as well as those headed by women or older people, are the most vulnerable to extreme weather events ^53^. The projection of the regional impacts of climate change on agricultural productivity in Brazil between 2021 and 2050 indicates that the Northeast Region will be the most affected due to its adverse weather conditions typical of a semiarid climate. Moreover, family farmers, who occupy 59% of the productive land in the North and Northeast regions, also tend to suffer greater impact due to their lower use of technology and greater dependence on favorable weather conditions ^58^.

The loss of productivity resulting from extreme weather events leads to a decline in the income of farmers and agricultural workers, while also reducing the availability of fresh foods, which is reflected in lower food supplies in local markets. In remote rural areas, in addition to poor infrastructure and sanitation, access to healthy food is also compromised, affecting the quantity and quality of food consumed on rural properties ^4^ ^,^ ^42^ ^,^ ^58^.

This situation can also result in an increase in unemployment, culminating in the displacement and migration of rural populations, who then concentrate in marginalized urban areas, thus increasing their exposure to extreme weather events ^4^ ^,^ ^42^ ^,^ ^54^. Greater urbanization contributes to an increase in temperature extremes and hinders rainfall runoff ^1^. Furthermore, the exodus of small producers favors the expansion of planting areas for large producers, who emit more GHG and require large quantities of water ^4^. This situation has been experienced in Brazil, which has seen its urban population reach its highest proportion (87.4%) in the last 22 years, while the rural population has reduced by 33.8%, which corresponds to around 25.6 million people (12.6% of the population) ^59^.

Therefore, the hegemonic food system fuels inequalities, which, in turn, ensure that the food system remains increasingly unfair, with a more substantial impact on specific populations (Figure 1, pathway (d.3), connection of vulnerability).

In rural areas, women are primarily responsible for ensuring water and food supplies within the household and also suffer disproportionately from the effects of droughts caused - or aggravated - by climate change ^4^. A greater distance required to fetch water increases the risk of various forms of gender-based violence (sexual violence, transactional sex, unintentional pregnancy, underaged marriages, etc.), which is a situation experienced by girls and women in the semiarid region (Figure 1, pathway (d.4), direct connection) ^60^. Pregnant women, nursing mothers, and children are even more sensitive to the risks related to extreme weather events due to their greater vulnerability to morbidities, such as infectious diseases, and greater nutritional needs ^61^.

### Impact of climate change and food insecurity on childhood malnutrition

Climate change impacts the health and nutritional status of children under five years of age both directly and indirectly. The direct impact refers to high temperatures, which increase the risk of miscarriage, and greater exposure to air pollutants and allergens, which increases the risk of respiratory infections and asthma in childhood, premature birth, low birth weight, and delayed child development ^62^ ^,^ ^63^ ^,^ ^64^ ^,^ ^65^. The indirect impact is mediated by food insecurity and manifests itself in different ways. In general, severe food insecurity and hunger are associated with undernutrition, while mild and moderate food insecurity are, paradoxically, associated with obesity ^2^.

As stated above, the interrelationship among the pandemics of undernutrition, obesity, and climate change is established in the global syndemic model. These pandemics interact with each other and share common social determinants, including the hegemonic food system and the concentration of power and wealth among a small number of corporations ^2^. Such characteristics connect to the model discussed in this essay by addressing social inequalities and the hegemonic food system as determinants of childhood malnutrition.

This understanding also aligns with the conceptual framework proposed by United Nations Children’s Fund (UNICEF) ^23^, which hierarchizes the determinants of maternal and child nutrition into three levels: on a broad level, facilitating determinants refer to political situations as well as environmental, social, and economic resources, and are connected to the dimension of social inequalities in the model discussed here; underlying determinants are linked to food and nutrition services and practices in homes, communities, and food environments, reflecting the dimensions of food insecurity, water insecurity, and food systems; immediate determinants involve healthy eating and adequate services and practices for good nutrition, which can prevent the manifestations of childhood malnutrition ^23^.

Evidence of these interrelationships has been published in recent years ^34^ ^,^ ^66^ ^,^ ^67^. Prolonged periods of drought are associated with impacts on the production and access to food by Indigenous women in rural Africa, leading to premature births ^66^. Extreme droughts or floods have been associated with lower linear growth and insufficient weight gain in children under five years of age in Nepal and Uganda due to diminished food production ^34^. Moreover, fetal and childhood undernutrition resulting from this context tends to increase the risk of obesity in later childhood and adulthood ^67^ (Figure 1, pathway (e.1), direct link).

Children with excess weight are more susceptible to heat-related illnesses and uncomfortable weather can lead to an even greater reduction in physical activity ^68^ ^,^ ^69^. Moreover, the increase in the frequency of extreme weather events reduces the production of fruits and vegetables, raising the prices of these items. In contrast, ultra-processed products remain widely available, as they depend on more structured agricultural chains and are less vulnerable to such events ^2^. Consequently, the price of ultra-processed foods tends to be lower, with a preference for the consumption of these products, mainly by families with a lower income and in a situation of food insecurity ^2^ ^,^ ^37^. Moreover, the growing proportion of the population with excess weight tends to increase food and beverage consumption due to greater metabolic demands, thus requiring an increase in agricultural production and, consequently, GHG emissions ^69^.

This scenario has also been growing in Brazil. The prevalence of excess weight among children under five years of age increased by four percentage points between 2006 and 2019 (6% vs. 10.1%) ^70^. Approximately 80% of these children frequently consume ultra-processed foods, such as cookies, crackers, and sugary drinks ^71^. In the lower-income population, this pattern may reflect the lack of physical and financial access to quality food ^70^ ^,^ ^72^.

Obesity can be prevented by healthy feeding practices in the first years of life, such as breastfeeding and adequate complementary feeding ^73^. Extreme weather events, however, can precipitate the interruption of breastfeeding due to a lack of support and private, safe breastfeeding environments as well as a lack of knowledge on strategies for its maintenance by mothers or caregivers ^74^ ^,^ ^75^. In 2019, the prevalence of continued breastfeeding up to one year of age was 52.1% in Brazil ^76^, which falls short of the target rate ^76^. Infant formulas, in turn, generate waste and are at risk of diminished availability in the occurrence of extreme weather events, in addition to constituting yet another potential source of contaminated water ^62^ ^,^ ^75^.

Low access to safe drinking water (measured by the water insecurity indicator) is also associated with a higher risk of undernutrition, as water is used for the washing of foods and meal preparation. Water contamination favors the incidence of diarrhea, which compromises child growth and development and constitutes one of the main causes of mortality in this age group ^77^ (Figure 1, pathway (e.3), direct connection).

Climatic phenomena, such as El Niño and La Niña, and higher temperatures are associated with reduced food diversity - one of the indicators of adequate complementary feeding ^78^ - among children between 9 and 36 months of age, compromising the quality and quantity of their diet ^33^. In Brazil, children 6 to 11 months of age were reported to be the group that consumed the least fruits and vegetables (25% non-consumption rate) and whose diet had the lowest minimum dietary diversity (46.8%) ^71^.

Low dietary diversity is linked to low micronutrient intake, increasing the risk of deficiencies and vulnerability to water-borne and food-borne infectious diseases. Children with anemia are more susceptible to malaria infection, the prevalence of which increases with climate change. However, iron supplementation used for treatment can aggravate the infection ^79^. Vitamin A deficiency aggravates damage to the intestinal mucosa and increases the mortality rate due to diarrhea, while vitamin D deficiency is associated with a greater risk of obesity and stunted growth ^80^ ^,^ ^81^.

In 2019, vitamin B12 (14.2%) and zinc (17.8%) deficiencies were most prevalent among Brazilian children 6 to 59 months of age, with lower rates of iron deficiency anemia (3.5%), vitamin A deficiency (6%) and vitamin D insufficiency (4.3%) ^82^. In this same population, 47.1% of children lived in families with some degree of food insecurity, with 3.8% in a situation of severe food insecurity ^83^, highlighting the relationship between food insecurity and malnutrition in childhood ^6^.

Thus, extreme weather events can cause malnutrition among young children, which may make them more vulnerable to climate change, while pre-existing malnutrition may increase the vulnerability of the health and nutritional status of these children to extreme weather events (Figure 1, pathway (e.2), connection of vulnerability).

As a reflection of these inequalities, malnutrition among Brazilian children is more concentrated in certain regions and specific populations, such as the North Region and among black or mixed-race children ^13^ ^,^ ^71^ ^,^ ^82^. Therefore, one can infer that children residing in poorer households, especially girls, black and Indigenous children, those belonging to *Quilombola* communities or other traditional peoples and communities, or socially excluded groups are at increased risk of malnutrition if they live in the context of climate change (Figure 1, pathway (e.4), direct connection).

This scenario can generate additional pressure on health and social protection systems, further impacting the rights of children to adequate food and health.

## Final considerations

The conceptual model proposed in this paper contributes to the understanding of the interactions among climate change, food insecurity, and children’s health/nutrition, and how these are aggravated by water insecurity and social inequalities. The model also highlights the role of Brazil’s hegemonic food system, which intensifies climate change as well as food insecurity and inequalities. Moreover, this model contributes to clarifying gaps in studies on climate change by approaching food insecurity from the perspective of access to food, as measured by the EBIA, and by recognizing social inequalities as central determinants of these relationships.

Thus, the conceptual model identifies vulnerability pathways and opportunities for intervention and can guide future studies aimed at ensuring adequate health and nutrition for Brazilian children in the context of climate change. This model can also favor the formulation, implementation, and monitoring of public policies with coordinated multisectoral actions for the adaptation to and mitigation of climate change in Brazil.

## Data Availability

Not applicable.
