# Supplementary material for: Climate change, food insecurity, and the impacts on child health and nutrition in Brazil: proposal for a conceptual model
Source: Cad Saude Publica. 2025 Dec 1;41(11):e00217824. doi: 10.1590/0102-311XEN217824 (PMC12688212; doi:10.1590/0102-311XEN217824)
Supplement: Translation

# Translation: Mudanças climáticas, insegurança alimentar e seus impactos na saúde e nutrição infantil no Brasil: proposta de modelo conceitual

**DOI:** 10.1590/0102-311XPT217824

**Authors:** Lissandra Amorim Santos-Degner, Poliana de Araújo Palmeira, Elisabetta Gioconda Iole Giovanna Recine, Elaine Martins Pasquim, Rosana Salles-Costa, Ana Maria Segall-Corrêa, Janaína Braga de Paiva, Larissa Ferreira Tavares Nonato, Sandra Maria Chaves dos Santos

**Affiliations:** 1 Universidade Federal da Bahia, Salvador, Brasil.; 2 Universidade Federal de Campina Grande, Campina Grande, Brasil.; 3 Universidade de Brasília, Brasília, Brasil.; 4 Ministério da Ciência, Tecnologia, Inovação e Comunicações, Brasília, Brasil.; 5 Instituto de Nutrição Josué de Castro, Universidade Federal do Rio de Janeiro, Rio de Janeiro, Brasil.; 6 Faculdade de Ciências Médicas, Universidade Estadual de Campinas, Campinas, Brasil.; 7 Centro de Ciências da Saúde, Universidade Federal da Paraíba, João Pessoa, Brasil.

**Keywords:** Mudanças Climáticas, Desnutrição Infantil, Insegurança Alimentar, Sistema Alimentar, Sindemia

## Abstract

As mudanças climáticas vêm causando maior frequência e intensidade dos eventos climáticos extremos, levando cerca de 72 milhões de pessoas em todo o mundo a enfrentar limitações no acesso aos alimentos em 2023. No Brasil, esse cenário agrava as situações de fome crônica, pobreza e desigualdades sociais, sobretudo, aumentando a vulnerabilidade em crianças menores. Este estudo tem por objetivo propor um modelo conceitual que explicite e explique estas relações. Foi realizada pesquisa bibliográfica com os termos “mudança climática”, “insegurança alimentar” e “má nutrição infantil”, e a combinação entre eles com a inclusão da palavra “Brasil”, no sentido de discutir o modelo no contexto da realidade brasileira. Foi proposto modelo conceitual que apresenta as relações entre os elementos principais e outros três de mediação e/ou complexidade - sistema alimentar, insegurança hídrica e desigualdades sociais - que se relacionam por cinco vias: (a) impacto direto dos eventos climáticos extremos sobre o acesso ao alimento; (b) impacto dos eventos climáticos extremos no acesso ao alimento, em decorrência dos seus efeitos no sistema alimentar; (c) insegurança hídrica como elemento que adiciona complexidade à relação dos extremos com a insegurança alimentar; (d) as desigualdades sociais como determinantes do efeito das mudanças climáticas sobre os domicílios em condição de insegurança alimentar, baixo acesso à água e/ou má nutrição infantil; (e) a saúde e nutrição infantil afetada por todos esses fatores. As conexões discutidas no modelo poderão orientar futuras pesquisas, favorecendo o desenvolvimento e a implementação de ações colaborativas e multissetoriais de adaptação e construção de resiliência às mudanças climáticas no Brasil.

## Introdução

O aumento da temperatura global é uma realidade e a cada ano se aproxima dos críticos 1,5℃ acima dos níveis do período pré-industrial (1850-1900), apontados pelo Acordo de Paris e pelos relatórios do Painel Intergovernamental de Mudanças Climáticas (IPCC, acrônimo em inglês). Junto com a maior frequência e intensidade dos eventos climáticos extremos, o aquecimento global compõe o que se denomina mudanças climáticas ^1^. Esse conjunto de alterações no clima tem uma natureza dinâmica e complexa que aponta para uma rápida ascensão e impacto catastrófico nos biomas e na alimentação e saúde humana, sendo, por isso, considerado uma pandemia ^2^. 

Um dos impactos mais significativos das mudanças climáticas ocorre na produção de alimentos e, aliados à escassez hídrica, à vulnerabilidade socioeconômica e baixa resiliência de algumas populações e à insuficiência de políticas públicas de adaptação e mitigação, têm agravado a fome ao redor do mundo ^3^ ^,^ ^4^ ^,^ ^5^ ^,^ ^6^ ^,^ ^7^ ^,^ ^8^. Globalmente, em 2023, as mudanças climáticas expuseram cerca de 72 milhões de pessoas à insegurança alimentar devido à limitação no acesso aos alimentos ^9^. Consequentemente, as prevalências de todas as formas de má nutrição - incluindo a desnutrição e o sobrepeso/obesidade - vêm crescendo em todo o mundo. 

A interação entre as pandemias, como no caso das mudanças climáticas, desnutrição e obesidade, é conceituada como uma sindemia global, afetando parcela expressiva da população mundial simultaneamente. Nesse contexto, a saúde das crianças menores de cinco anos tem risco particularmente maior devido à imaturidade fisiológica desta fase e à elevada demanda de nutrientes ^2^. Mundialmente, em 2022, cerca de 148,1 milhões (22,3%) de crianças nessa faixa etária tiveram déficit de estatura, enquanto o baixo peso para a estatura alcançou 5 milhões (6,8%) e 37 milhões (5,6%) o excesso de peso ^10^. 

No Brasil, as taxas de má nutrição infantil refletem a maior prevalência e gravidade de insegurança alimentar existente nos domicílios com a presença de crianças ^11^ ^,^ ^12^. Em 2019, o déficit de estatura atingiu 7% das crianças brasileiras com menos de cinco anos, o baixo peso para idade alcançou 2,9% delas e 10,1% o excesso de peso ^13^. A coexistência da fome, enquanto problema político-social, com a desnutrição e a obesidade, evidenciam o caráter preocupante da sindemia no Brasil. Esse contexto se soma às desigualdades sociais estruturais no país e determinam a distribuição e gravidade da insegurança alimentar nos diversos domicílios brasileiros ^11^ ^,^ ^12^ ^,^ ^14^ ^,^ ^15^ ^,^ ^16^. 

Logo, há o desafio de esclarecer quais relações podem ser estabelecidas entre os diferentes eventos climáticos extremos e seus impactos no direito humano à alimentação e na saúde e nutrição infantil. Alguns estudos abordam as conexões entre as mudanças climáticas e seus efeitos na saúde e nutrição humana ^17^ ^,^ ^18^. No entanto, persistem lacunas na abordagem teórico-científica sobre as conexões destes aspectos com as desigualdades sociais e a experiência de fome e insegurança alimentar. 

Considerando o contexto histórico de desigualdades sociais e as possibilidades de agravamento da má nutrição infantil, o objetivo deste ensaio é propor um modelo conceitual que contribua para o debate das relações entre mudanças climáticas, com seus eventos climáticos extremos, a insegurança alimentar e a saúde e estado nutricional das crianças brasileiras.

## Métodos

Este ensaio integra e subsidia um estudo maior, intitulado *Indicadores de Mudanças Climáticas, de Insegurança Alimentar e Fome no Brasil e seus Impactos na Saúde e Nutrição Infantil*. Ele reúne pesquisadores de diferentes instituições, com o objetivo de desenvolver a plataforma FomeS (https://fomes.net.br/), composta por indicadores construídos a partir de bases de dados nacionais públicas. A plataforma será destinada ao uso pela comunidade científico-institucional e pelos movimentos sociais interessados, fomentando o preenchimento de lacunas científicas sobre as dimensões abordadas.

O modelo conceitual desenvolvido caracteriza a primeira etapa do estudo e se propôs a estabelecer as suas referências iniciais, a partir de uma revisão da literatura para descrever o conhecimento existente e identificar as lacunas sobre o tema ^19^. No sentido de recompor, no plano conceitual, as complexas relações que se dão entre as dimensões das mudanças climáticas, insegurança alimentar e má nutrição infantil no mundo real, esse modelo representa o ponto de partida para a identificação de outras dimensões relacionadas e a seleção dos indicadores que irão compor a plataforma FomeS.

### Estratégias de busca bibliográfica

O primeiro passo consistiu na pesquisa bibliográfica, realizada em bases de dados previamente selecionadas (PubMed, Web of Science e SciELO), com termos indexadores em inglês, espanhol ou português, no período de fevereiro a agosto de 2024. A estratégia de busca foi a combinação de três grandes blocos de termos de investigação: saúde e nutrição das crianças, segurança alimentar/insegurança alimentar e mudanças climáticas ou eventos climáticos extremos (como ondas de calor extremo, secas, incêndios florestais e inundações). A palavra “Brasil” foi combinada com cada um desses blocos, no intuito de discutir o modelo proposto no contexto brasileiro. Selecionou-se relatórios, boletins e estudos técnicos, além de artigos revisados por pares de diferentes países, totalizando 150 documentos, cujos conteúdos mais relevantes foram utilizados para a construção do modelo.

### Sistematização e elaboração do modelo conceitual

Para sistematização, usou-se o termo “segurança alimentar” a partir de perspectivas conceituais diversas, considerando volume e produção agrícola, mas também abordagens relacionadas à disponibilidade de alimentos, acesso, consumo e utilização biológica dos alimentos pelos indivíduos. Os estudos encontrados abordam o impacto das mudanças climáticas sobre a segurança alimentar e nutricional e foram realizados em pequenos grupos de pessoas ou com dados de inquéritos nacionais, principalmente em países da Ásia e África, sob a perspectiva da produção ou do consumo de alimentos. Não foram encontrados estudos empíricos no Brasil, sendo sistematizados para discussão dois estudos com projeções sobre o possível impacto das mudanças climáticas na produção nacional de alimentos (Quadro 1).

**Quadro 1:** Estudos selecionados sobre a relação entre mudanças climáticas e segurança/insegurança alimentar, segundo país.

PAÍS	EVENTO CLIMÁTICO EXTREMO	DIMENSÕES DA SEGURANÇA ALIMENTAR	MÉTODO/CATEGORIAS DE ANÁLISE	ESTUDO (ANO)
Argentina, Cazaquistão, Marrocos e África do Sul	Variações de temperatura, intensidade das chuvas e da seca	Produção de alimentos	Análise comparativa dos registros de produção de cereais observados com distribuições contrafactuais e factuais estimadas para a produtividade agrícola, segundo regiões e ocorrência de eventos climáticos extremos e em longo prazo	Organização das Nações Unidas para a Alimentação e a Agricultura ^4^ (2023)
Brasil	Mudanças climáticas projetadas	Produção de alimentos	Análise do impacto das mudanças climáticas projetadas sobre os polinizadores de 13 culturas, usando técnica computacional para determinar áreas potenciais de ocorrências de espécies e prever sua distribuição futura	Giannini et al. ^84^ (2017)
Brasil	Dados projetados de temperatura e precipitação, segundo cenários propostos pelo IPCC (RCP 4.5 e RCP 8.5)	Produção de alimentos	Estimação em *cross section* de uma função de produção em que os resultados, em termos de produtividade agrícola, são determinados por fatores climáticos, geográficos e produtivos	Tnure et al. ^58^ (2024)
China	Temperatura média diária e variações de temperatura, precipitação, velocidade média do vento, umidade relativa e evaporação	Produção de alimentos Disponibilidade e consumo de alimentos	Segurança alimentar nacional medida em cinco grupos de indicadores: segurança quantitativa (produção de grãos); segurança qualitativa (uso de fertilizantes e pesticidas); segurança ambiental ecológica (taxa de desastre de grãos); segurança econômica (preço) e segurança de recurso (área de plantio e água para produção de grãos)	Zhou et al. ^26^ (2024)
México	Chuvas e tempestades	Produção de alimentos	Avaliação da variabilidade da precipitação e os rendimentos de milho no México em três períodos de tempo diferentes: o presente, os últimos 30 anos e cenários futuros de RCPs	Murray-Tortarolo et al. ^29^ (2018)
Nepal e Uganda	Variações mensais de precipitação	Utilização biológica dos alimentos	Avaliação do crescimento linear e ganho de peso de crianças com menos de 5 anos, com a variação de precipitação durante períodos chave da fase.	Shively ^34^ (2017)
Peru (Iquitos, Loreto)	El Niño Oscilação Sul (ENOS)	Consumo de alimentos	Estudo observacional longitudinal de coorte (2010-2014) com crianças de 9 a 36 meses e aplicação de recordatório de 24 horas	Ambikapathi et al. ^33^ (2021)
Uganda	Seca extrema prolongada, ondas de calor, estações irregulares	Produção de alimentos Disponibilidade e consumo de alimentos	Estudo qualitativo com mulheres indígenas e não indígenas de áreas rurais de Uganda	Bryson et al. ^66^ (2021)
Timor Leste, Nepal, Filipinas, Nigéria, Libéria, Gana, Madagascar, Uganda, Zâmbia, Zimbábue, Namíbia, Suazilândia, Lesoto, Egito, Peru, Guiana, Colômbia, República Dominicana e Haiti	Variações de temperatura e precipitação	Disponibilidade e consumo de alimentos	Dados de pesquisas de 19 países - diversidade dietética infantil 30 anos de dados de clima	Niles et al. ^85^ (2021)

IPCC: *Intergovernmental Panel on Climate Change* (Painel Intergovernamental sobre Mudanças Climáticas); RCP: *representative concentration pathways* (caminhos de concentração representativos).

O conceito brasileiro de segurança alimentar e nutricional cunhado em 2006 ^20^, foi utilizado como guia para o debate. Os termos “segurança alimentar” e “insegurança alimentar” foram utilizados em referência à classificação de indicador da situação de acesso ou privação alimentar de famílias, resultante da aplicação da *Escala Brasileira de Insegurança Alimentar* (EBIA), instrumento de mensuração da fome e insegurança alimentar no país ^21^ ^,^ ^22^. Com base na EBIA, quando a família tem acesso regular e permanente a alimentos, em qualidade e quantidade suficientes, considera-se presença de segurança alimentar. A insegurança alimentar, por sua vez, evidencia a privação do acesso aos alimentos e é relatada em três níveis: leve, moderado ou grave, sendo este último uma condição em que a fome é uma experiência vivida no domicílio, inclusive pelas crianças ^22^.

### Validação interna do conteúdo do modelo conceitual

Visando o consenso dos parâmetros teóricos desenvolvidos na elaboração do modelo conceitual proposto, realizou-se uma oficina virtual com os membros da equipe. A oficina foi desenvolvida em três etapas: (i) envio de link com o referencial teórico para leitura e comentários prévios ao encontro; (ii) apresentação sistematizada do referencial teórico e da figura do modelo conceitual; e (iii) diálogo entre os pesquisadores participantes com ajustes no modelo. Participaram da atividade 11 integrantes, sendo três pesquisadores da área da Ciência de Dados e oito da área da Saúde e Segurança Alimentar e Nutricional. A oficina garantiu o diálogo entre a equipe, o que culminou na validação de uma proposta de modelo conceitual, em que foram consideradas as dimensões identificadas e suas definições, bem como as inter-relações entre seus diversos elementos e um glossário.

## Resultados

Os termos que nortearam a construção do modelo conceitual proposto estão sistematizados em um glossário (Quadro 2). O modelo se baseia na abordagem de sistemas complexos que reconhecem a interconexão entre os elementos de um sistema, sendo que realizar alterações em um deles gera impacto nos meios interno e externo. Assim, em vez de analisar partes isoladas, essa abordagem busca compreender o todo, considerando as relações, interações e emergências resultantes ^17^ ^,^ ^23^. 

**Quadro 2:** Glossário dos termos utilizados no modelo conceitual das inter-relações entre eventos climáticos extremos, insegurança alimentar e má nutrição infantil.

TERMOS	DEFINIÇÃO		
Aquecimento global	“*É o processo de aumento da temperatura próximo à superfície do planeta causado pela presença de gases causadores do efeito estufa*” ^86^ (p. 3)		
Bioma	“*São agrupamentos de ecossistemas de tipos de vegetação vizinhos que apresentam condições geográficas e climáticas semelhantes. No Brasil, são seis biomas: Amazônia, Caatinga, Cerrado, Mata Atlântica, Pampa e Pantanal. Há ainda o Sistema Costeiro-Marinho abrangendo o litoral e as águas territoriais considerado o 7º bioma brasileiro*” ^86^ (p. 4)		
Clima	“*É geralmente definido como a média regional ou mesmo global de longo prazo (normalmente a média é de 30 anos) de temperatura, umidade e padrões de precipitação e vento ao longo de um período de tempo que varia de meses ou estações a milhares ou milhões de anos*” ^36^ (p. 544).		
Desigualdades sociais	“*São diferenças sistemáticas e persistentes de acesso a bens, recursos e oportunidades, que se estabelecem entre pessoas, grupos sociais ou mesmo populações inteiras, geradas independentemente dos talentos, capacidades e desempenhos individuais. As desigualdades sociais podem ser de classe, étnico-raciais ou de gênero*” ^87^ (p. 298)		
Desmatamento	“*Conversão da vegetação nativa para pastagens e cultivos agrícolas*” ^88^ (p. 46)		
Gases de efeito estufa	“*São constituintes gasosos da atmosfera (H*_*2*_ *O, CO*_*2*_ *, N*_*2*_ *O , CH*_*4*_ *e O*_*3*_ *, dentre outros), tanto naturais quanto antropogênicos, que absorvem e emitem radiação dentro do espectro da radiação infravermelha térmica emitida pela superfície da Terra, pela própria atmosfera e pelas nuvens*” ^36^ (p. 550-1)		
Efeito estufa	“*Fenômeno natural que garante a temperatura adequada para a vida na terra, ocasionado pela concentração de gases na atmosfera que formam uma camada para passagem dos raios solares e a absorção de calor. O aumento da concentração desses gases espessa a camada dificultando a dispersão da radiação solar e provocando maior retenção de calor* ^86^ (p. 8)		
El Niño e La Niña	“*Fenômenos climáticos caracterizados pelo aquecimento (El Niño) ou resfriamento (La Niña) da superfície do Oceano Pacífico, que causam alterações nos padrões de circulação atmosférica em todo o planeta*” ^86^ (p. 8)		
*Escala Brasileira de Insegurança Alimentar* (EBIA)	Escala psicométrica brasileira de medida direta da insegurança alimentar, a partir da experiência vivenciada e percebida pelas pessoas afetadas, residentes do domicílio ^22^		
Eventos climáticos extremos	“*Refere-se a ocorrência de um valor de uma variável climática ou de tempo acima (ou abaixo) de um valor limite próximo às extremidades superior (ou inferior) do intervalo de valores observados da variável (inclui ondas de calor extremo, secas, incêndios florestais, inundações, entre outros)*” ^86^ (p. 9)		
Insegurança alimentar	Privação no acesso regular e permanente a alimentos de qualidade e em quantidade suficiente nas famílias. A partir da percepção da experiência do domicílio nos últimos 90 dias, a EBIA aponta para um dos seguintes níveis da insegurança alimentar vivida pelas famílias: leve, moderada ou grave ^22^		
Insegurança hídrica domiciliar	“*Definida como a incapacidade de acessar e se beneficiar de água de qualidade adequada e em quantidade suficiente para todos os usos domésticos, humano e produção, quando aplicável. Isto é, confiável (do ponto de vista da fonte) e segura (do ponto de vista da qualidade sanitária) para o bem-estar e uma vida saudável*” ^89^ (p. 2)		
Má nutrição infantil	Condição que se manifesta devido ao desequilíbrio de nutrientes essenciais para o crescimento e desenvolvimento, e ocorre quando a alimentação de uma criança não tem a quantidade adequada de nutrientes e energia. Inclui todas as formas de má nutrição (desnutrição por baixo peso para estatura, déficit de estatura ou baixo peso para idade; a deficiência de vitaminas ou minerais, o sobrepeso, obesidade e doenças não transmissíveis relacionadas à dieta) ^23^		
Mudanças climáticas	“*Qualquer mudança no clima ao longo dos anos, devido à variabilidade natural e/ou como resultado da atividade humana. Inclui o aumento da temperatura da superfície global, alterações de precipitação, umidade e vento, com maior ocorrência de eventos climáticos extremos* ^36^ (p. 544)		
*Representative concentration pathways* (RCPs)	Em português, “caminhos de concentração representativos”. “*São trajetórias de concentrações de gases de efeito estufa usadas para modelar o clima. O IPCC* [Painel Intergovernamental sobre Mudança do Clima] *usa os RCPs para criar cenários futuros de mudança climática*” ^36^ (p. 556)		
Saúde e nutrição infantil	Promoção de hábitos alimentares saudáveis, e prevenção da má nutrição infantil em todas as suas formas, a partir da abordagem sistêmica à nutrição. Esta abordagem fortalece a capacidade de cinco sistemas principais - alimentação, saúde, água e saneamento, educação e proteção social - de fornecer dietas, serviços e práticas que apoiem a nutrição materna e infantil adequada ^23^		
Segurança alimentar	Quando todas as pessoas, em todos os momentos, têm acesso físico, social e econômico a alimentos suficientes, seguros e nutritivos que atendam às suas necessidades dietéticas e preferências alimentares para uma vida ativa e saudável ^51^		
Segurança alimentar e nutricional	Consiste na realização do direito de todos ao acesso regular e permanente a alimentos de qualidade, em quantidade suficiente, sem comprometer o acesso a outras necessidades essenciais, tendo como base práticas alimentares promotoras de saúde que respeitem a diversidade cultural e que sejam ambiental, cultural, econômica e socialmente sustentáveis ^22^		
Segurança hídrica	Disponibilidade de água em quantidade e qualidade suficiente para o atendimento às necessidades humanas, à prática das atividades econômicas e à conservação dos ecossistemas aquáticos, acompanhada de um nível aceitável de risco relacionado a secas e cheias ^90^		
Sindemia global de obesidade, desnutrição e mudanças climáticas	“*Se refere à sinergia entre as pandemias de obesidade, desnutrição e mudanças climáticas que interagem entre si, partilhando fatores sociais subjacentes comuns e produzindo consequências complexas que se interrelacionam*” ^2^ (p. 794-5)		
Sistema alimentar	“*Conjunto de todos os elementos (ambiente, pessoas, insumos, processos, infraestruturas, instituições etc.) e atividades que se relacionam com a produção, processamento, distribuição, preparação e consumo de alimentos, e o resultado dessas atividades, incluindo socioeconômicos e ambientais. O sistema alimentar inclui três subsistemas: (i) cadeia de abastecimento de alimentos (abrange atividades como produção, armazenamento, distribuição, processamento, embalagem, varejo e marketing); (ii) ambiente alimentar (engloba dimensões da disponibilidade, como preços, propriedades do fornecedor e do produto e pessoais, como acessibilidade e conveniência de fontes de alimentos e produtos); e (iii) comportamento alimentar (reflete as escolhas e decisões feitas pelo consumidor em relação a qual alimento escolher e como estocar, preparar e consumir o alimento dentro do domicílio)*” ^91^ (p. 23)		

O modelo aponta caminhos da relação entre os eventos climáticos extremos e a má nutrição infantil, mediada pela condição de insegurança alimentar e outros fatores, como o sistema alimentar, a insegurança hídrica e as desigualdades sociais (Figura 1). As linhas sólidas representam as conexões diretas (vias a, b.2, c.1, d.2, e.1, e.3 e e.4); as linhas pontilhadas indicam conexões de reciprocidade (vias b.1, c.2 e d.1); as linhas tracejadas evidenciam conexões de coexistência (c.3); enquanto as linhas com traços e pontos representam as conexões que aumentam a vulnerabilidade aos eventos climáticos extremos (d.3, d.4 e e.2). 

**Figura 1:** Modelo conceitual indicando os caminhos que as mudanças climáticas, através dos eventos climáticos extremos, percorrem para impactar a saúde e nutrição infantil, tendo a insegurança alimentar como mediadora.

Nota: letras (a-e) indicam as diferentes dimensões; números (1-4) indicam as diferentes vias de conexão entre as dimensões. Via (a), impacto direto das mudanças climáticas sobre a insegurança alimentar; vias (b) (b.1 e b.2), relação do sistema alimentar com as mudanças climáticas e a insegurança alimentar; vias (c) (c.1 a c.3), relação da insegurança hídrica com os demais componentes; vias (d) (d.1 a d.4) mostram como as desigualdades sociais determinam as relações; e vias (e) (e.1 a e.4), como a má nutrição sofre influência da insegurança alimentar, mudanças climáticas, insegurança hídrica e desigualdades sociais.

Tomando a insegurança alimentar como dimensão central, definiram-se outras cinco dimensões (a, b, c, d, e) e as vias de conexão entre elas (1 a 4). A via (a) considera o impacto emergencial aos eventos climáticos extremos no acesso aos alimentos, em um momento específico, independentemente da condição prévia de insegurança alimentar. As vias b.1 e b.2 consideram o papel mediador do sistema alimentar na relação entre as mudanças climáticas e a insegurança alimentar. Esta conexão apresenta um nível de complexidade maior quando a insegurança alimentar se associa à insegurança hídrica (vias c.1/c.2/c.3). As desigualdades sociais (d) adicionam outra camada de complexidade ao determinarem a forma como os eventos climáticos extremos afetam as demais dimensões (vias d.1/d.2/d.3/d.4). Enquanto as últimas (vias e.1/e.2/e.3/e.4), apresentam como a saúde e nutrição infantil são impactadas pelos eventos climáticos extremos, insegurança alimentar, insegurança hídrica e desigualdades, gerando ou agravando a má nutrição entre as crianças menores de cinco anos.

## Discussão

### Impacto das mudanças climáticas na insegurança alimentar

Nos últimos cinco anos, secas e inundações levaram 40% dos municípios brasileiros a estados de emergência, o que tem impulsionado a migração interna por desastres climáticos, resultando nos maiores índices da América Latina em 2023 ^24^ ^,^ ^25^. Esse contexto agrava a privação alimentar, independentemente da condição de insegurança alimentar anterior (Figura 1, via (a), conexão direta). 

Estudos que avaliam o impacto das mudanças climáticas na insegurança alimentar ainda são escassos, especialmente considerando como indicadores as escalas de experiência de fome. Estudo realizado na China ^26^ mostrou que a temperatura apresentou um efeito linear na segurança alimentar, ou seja, quanto maior a temperatura, maior o impacto negativo neste indicador. Por outro lado, o estudo indicou que a precipitação demonstrou influência de padrão não linear, isto é, contribui positivamente para os indicadores de segurança alimentar da população estudada dentro de um intervalo ótimo, enquanto o aumento acima deste intervalo culminaria em impacto negativo. Ressalta-se que o conceito de segurança alimentar na China, diferentemente do Brasil, está mais relacionado a indicadores de produção de alimentos ^26^. 

Ainda na década de 1940, Josué de Castro descreveu a ocorrência da fome epidêmica na região do sertão nordestino como um fenômeno consequente às secas cíclicas e extremas da região, em um cenário de ausência do Estado e de políticas públicas ^27^. No entanto, até o momento, não foram identificados estudos que avaliaram o efeito das mudanças climáticas na insegurança alimentar e na fome no Brasil, a partir do uso da EBIA como um dos indicadores. O aumento da ocorrência de eventos climáticos extremos alerta não apenas para a dificuldade de acesso aos alimentos, quando há impactos na produção com redução de culturas agrícolas, mas para o comprometimento imediato da renda e exposição aguda à insegurança alimentar nas comunidades atingidas, com consequências emergenciais e de longo prazo, principalmente entre as famílias mais vulneráveis ^9^ ^,^ ^28^.

### Sistemas alimentares

As mudanças climáticas podem impactar a segurança alimentar por diversas vias, sendo os sistemas alimentares uma das principais ^8^. Fatores como o declínio dos insetos polinizadores, a proliferação de pragas e patógenos, aumento da escassez de água potável e as mudanças das concentrações de gases e minerais na atmosfera e no solo representam consequências das mudanças climáticas envolvidas no comprometimento da produção dos alimentos, reduzindo a quantidade produzida e o seu conteúdo nutricional ^5^ ^,^ ^6^ ^,^ ^7^ ^,^ ^8^. 

Globalmente, estima-se que, nos últimos trinta anos, os extremos climáticos levaram à perda de produtos agrícolas que, se convertidos em nutrientes, equivalem às necessidades alimentares diárias de aproximadamente 450 milhões de indivíduos ^4^. As reduções de micronutrientes nos alimentos parecem ser particularmente expressivas para o ferro, o fósforo, o magnésio e a tiamina, atingindo mais o grupo dos cereais, mas também as culturas de frutas e hortaliças e cana de açúcar ^4^ ^,^ ^7^. 

Há evidências da redução de 2% até 25% na quantidade produzida de cereais (trigo e milho) em países como Cazaquistão, Marrocos, África do Sul e México devido aos eventos climáticos extremos ^4^ ^,^ ^29^. Na América Latina, a maior frequência das ondas de calor e meses de seca em 2021 foi associada ao aumento de 9,9 milhões de pessoas com menor acesso aos alimentos ^3^. Setores como o de pesca e aquicultura também registraram impactos como a redução do tamanho e alteração da composição de diversas espécies de peixes ^30^ e perda de biodiversidade ^31^. 

No Brasil, a safra nacional de grãos, cereais e leguminosas de 2024 sofreu queda de 7,2% devido aos eventos climáticos extremos ocorridos no ano anterior ^32^. Estudos de projeção estimaram uma redução de 3,1% na produtividade média das culturas agregadas a cada aumento de 1ºC na temperatura média durante as estações mais quentes do ano ^33^, além da redução de ocorrência de polinizadores em diversos municípios brasileiros ^34^. Essas previsões se aplicam a culturas de diversos alimentos, incluindo milho, feijão, café, goiaba, tomate, entre outros ^33^ ^,^ ^34^.

Ainda assim, o país vem se consolidando como um dos maiores produtores de alimentos no cenário mundial. O sistema alimentar hegemônico no Brasil, no entanto, é caracterizado pela produção em larga escala e mecanizada de alimentos, *commodities* ou não, com o predomínio da pecuária e monocultura e do uso intensivo de água e agrotóxicos. A distribuição globalizada dos alimentos envolve processamento, embalagem e transporte que exploram os recursos naturais e emitem maior quantidade de gases de efeito estufa (GEE) ^8^ ^,^ ^28^ ^,^ ^35^. Esse sistema favorece a produção e consumo de alimentos ultraprocessados, além de estimular a concentração de terras e recursos entre poucos e perpetuar a invisibilidade da cultura alimentar das populações tradicionais no Brasil. Esse cenário contribui para o agravamento das desigualdades sociais no país ^2^ ^,^ ^35^, além de ser o principal responsável pelas emissões brutas totais de GEE no país (73,7%) ^36^. 

Referente ao ambiente alimentar, destacam-se as desigualdades no acesso físico a alimentos adequados e saudáveis, em especial nas periferias urbanas do país. A distribuição desigual dos estabelecimentos comerciais que vendem alimentos saudáveis e não saudáveis contribui para aprofundar as desigualdades sociais, uma vez que regiões de maior vulnerabilidade frequentemente apresentam menor disponibilidade de feiras, de mercados locais e outros pontos de venda de alimentos *in natura* ou minimamente processados a preços acessíveis. Em contrapartida, observa-se nesses territórios a predominância de pequenos comércios e redes varejistas que priorizam a oferta de alimentos ultraprocessados, muitas vezes mais acessíveis economicamente devido a fatores que incluem a produção em larga escala, incentivos fiscais diferenciados e maior poder de negociação da indústria ^37^ ^,^ ^38^ ^,^ ^39^. 

Esse contexto se caracteriza como um dos fatores que contribui para o consumo crescente de alimentos ultraprocessados, fenômeno observado nos últimos dez anos no Brasil ^40^. Esse consumo teve crescimento mais expressivo entre a população com menor renda (3,4 pontos percentuais - p.p.) e indígena (5,96p.p.) ^40^. Adicionalmente, o maior consumo desses alimentos está inversamente associado à adesão a uma dieta saudável e sustentável ^41^. Logo, o sistema alimentar hegemônico pode ser considerado insustentável, injusto e promotor de hábitos alimentares e de vida não saudáveis ^2^ ^,^ ^8^ ^,^ ^35^. 

A relação entre as mudanças climáticas e o sistema alimentar, portanto, é bidirecional, de determinação mútua. Por um lado, as mudanças climáticas reduzem a produção de alimentos em volume e qualidade de nutrientes; por outro, o sistema alimentar hegemônico, tal como é, contribui para o agravamento delas (Figura 1, via (b.1), conexão de reciprocidade).

Cabe ainda considerar que a maior frequência e intensidade dos eventos climáticos extremos determina e agrava a situação de insegurança alimentar, na perspectiva do acesso ao alimento, em três aspectos: (i) instabilidade e redução da produção de alimentos, gerando menor rendimento para os pequenos produtores; (ii) tal redução influencia a dinâmica de mercado culminando em maior preço para o consumidor final, principalmente dos alimentos *in natura*; (iii) maior preço dos alimentos *in natura*, favorecendo o consumo dos alimentos ultraprocessados e, por consequência, a maior produção destes torna-os ainda mais baratos e acessíveis, diminuindo assim o acesso aos alimentos de qualidade (Figura 1, via (b.2), conexão direta).

### Insegurança hídrica

As mudanças climáticas comprometem o acesso e utilização de água potável por diversas vias, a saber: (i) a intrusão de água salgada nos aquíferos costeiros, devido às perdas glaciais e derretimento da neve; (ii) as inundações, que contaminam a água potável com patógenos causadores de infecções; e (iii) as secas frequentes e persistentes ^1^ ^,^ ^7^ ^,^ ^8^ ^,^ ^42^. A elevação da temperatura da superfície terrestre também altera o ciclo hídrico, culminando na escassez de água, que vem crescendo nas últimas décadas ^43^ (Figura 1, via (c.1), conexão direta). 

O desmatamento, resultado do sistema alimentar hegemônico, contribui para a tendência de aceleração do ciclo da água, que resulta no agravamento de secas e inundações ^44^. Os meios de produção, processamento e transporte dos alimentos, principalmente os ultraprocessados, utilizam elevados volumes de água e poluem os recursos hídricos disponíveis ^8^. Adicionalmente, o consumo de alimentos ultraprocessados e carne estão associados à maior pegada hídrica (aumento de 10,1% entre os quintis de consumo) ^43^ ^,^ ^45^ (Figura 1, via (c.2), conexão de reciprocidade). 

A escassez hídrica agravada pelos eventos climáticos extremos, por sua vez, tende a exercer pressão sobre o abastecimento alimentar, uma vez que cerca de 70% da água doce utilizada no mundo é destinada à agricultura ^1^ ^,^ ^46^. A redução da produtividade agrícola, por falta de água, afetaria a segurança alimentar local e, em contextos em que a agricultura é uma atividade de subsistência, a insegurança hídrica pode provocar compensações entre alocar água para fins agrícolas ou para o consumo ^8^ ^,^ ^47^ ^,^ ^48^ e precipitar movimentos de migração interna ^4^ ^,^ ^42^ (Figura 1, via (c.2)). 

Ainda que possua uma das maiores reservas de água doce do mundo, o Brasil também se encontra em risco de escassez hídrica por diversos fatores, independentemente das mudanças climáticas. A expansão da área de cultivo, que utiliza intensa irrigação, a variabilidade espacial das reservas de água - 70% da água doce disponível no país se encontra na Região Norte - e a crescente demanda de água decorrente do aumento populacional e processo de industrialização representam alguns deles ^49^. Além disso, o país tem regiões de alta vulnerabilidade ao aumento da temperatura da superfície, como o semiárido brasileiro, que tem como características a baixa taxa pluviométrica média anual e períodos de estiagem de até 11 meses. Esse cenário pode agravar o risco de desertificação da região, culminando, no futuro, em total inviabilidade das culturas de alimentos ^50^.

Em nível domiciliar, a insegurança hídrica compromete a higiene do domicílio, das pessoas, dos alimentos e preparo das refeições e amplia o risco de transmissão de doenças infecciosas ^51^. Dados nacionais do período entre o final de 2021 e o início de 2022 mostraram que cerca de 12% da população vivia com restrição de acesso à água e, destes, 65% também vivenciavam restrição quantitativa de alimentos (insegurança alimentar moderada ou grave), evidenciando a associação entre as duas inseguranças no Brasil ^52^. A conexão entre insegurança alimentar e insegurança hídrica, portanto, é de coexistência (Figura 1, via (c.3)). Ambas podem coexistir no mesmo ambiente e interagir entre si, compartilhando determinantes similares, relacionados com características socioeconômicas e demográficas ^47^.

### Desigualdades sociais

Diferenças em condições socioeconômicas, locais de moradia, acesso a instituições e serviços, bem como, normas sociais discriminatórias, determinam a vulnerabilidade climática de grupos sociais específicos, que se agrega à susceptibilidade pré-existente à insegurança alimentar e insegurança hídrica e ao ciclo de injustiça perpetuado pela dinâmica do sistema alimentar hegemônico ^53^. 

A vulnerabilidade climática seria a propensão ou predisposição de um indivíduo, ou grupo social, de ser afetado adversamente pelas mudanças climáticas, sendo resultado da intersecção de três fatores: exposição, susceptibilidade e capacidade adaptativa ^53^. Em uma situação de inundações, por exemplo, populações em situação de vulnerabilidade socioeconômica têm habitações precárias e/ou em locais de risco (maior exposição); essas condições os tornam mais suscetíveis aos impactos diretos da inundação, com maior probabilidade de perda de bens e moradia; enquanto a limitação de recursos financeiros reduziria sua capacidade de recuperação, retroalimentando o ciclo de vulnerabilidade ^5^ ^,^ ^53^. 

Assim, as desigualdades sociais tornam grupos sociais mais vulneráveis aos eventos climáticos extremos. Ao mesmo tempo, globalmente, as pessoas mais afetadas pela crise climática são as que menos contribuem para o aquecimento do planeta ^1^. Em 2019, aqueles que viviam na faixa de 1% mais ricos no mundo foram responsáveis por 16% das emissões globais de GEE, percentual que equivale às emissões de cerca de 5 bilhões de pessoas (66% da população global) ^5^ ^,^ ^53^ ^,^ ^54^ (Figura 1, via (d.1), conexão de reciprocidade). 

No Brasil, o cenário de desigualdades sociais tem raízes históricas e estrutura as bases de seu desenvolvimento. O modelo de produção alimentar atual se pauta - e sempre se pautou - na distribuição desigual de terras, alimentos, recursos e oportunidades, com concentração produtiva em poucos alimentos voltados à exportação e pouco apoio às pequenas propriedades rurais ^55^. Em consequência, ainda que o país produza alimento suficiente para alimentar cerca de 800 milhões de pessoas ^56^, a fome segue sendo um problema crônico que se concentra em regiões e grupos sociais específicos, incluindo a área rural (Figura 1, via (d.2), conexão direta).

As famílias que residem nas regiões Norte e Nordeste do Brasil são as mais atingidas pela insegurança alimentar. Em 2023, a restrição grave de acesso aos alimentos (fome) atingia 7,7% das famílias localizadas na Região Norte, proporção quase quatro vezes maior, comparado às famílias do Sul do país (2%) ^14^. Adicionalmente, a insegurança alimentar é mais prevalente nos lares chefiados por mulheres ^12^, por pessoas pretas ou pardas (69,6% *vs*. 29%) ^14^, entre as populações quilombolas (86,1% dos domicílios) ^16^ e os povos indígenas do Mato Grosso do Sul (76,7%) ^15^. 

A intersecção dessas características produz níveis mais profundos de vulnerabilidade climática. Famílias chefiadas por mulheres negras têm probabilidade quatro vezes maior de reportar insegurança alimentar moderada ou grave, quando comparados àquelas chefiadas por homens brancos ^12^. É conhecida a associação da condição de insegurança alimentar com outros indicadores de pobreza, como a baixa escolaridade, maior número de moradores nos domicílios e menor renda, o que reduz ainda mais as possibilidades dessas populações de quebrar o ciclo da pobreza e fome que vivenciam e, portanto, de lidar com os efeitos dos eventos climáticos extremos ^11^ ^,^ ^53^ ^,^ ^57^.

Ademais, famílias residentes da área rural também são mais vulneráveis à insegurança alimentar e aos eventos climáticos extremos. Dados nacionais de 2023 mostram que a proporção de insegurança alimentar grave na área rural foi 1,6p.p. superior ao verificado na área urbana (5,5% *vs*. 3,9%) ^14^. Nessas áreas, agricultores familiares e os domicílios com menor renda, além daqueles chefiados por mulheres ou pessoas idosas, representam os mais vulneráveis aos eventos climáticos extremos ^53^. A projeção dos impactos regionais das mudanças climáticas na produtividade agrícola no Brasil, entre 2021 e 2050, aponta a Região Nordeste como a mais afetada devido às suas condições climáticas adversas, típicas de um clima semiárido. Além disso, os agricultores familiares, que ocupam 59% das terras produtoras nas regiões Norte e Nordeste, também tendem a sofrer maior impacto devido ao menor uso de tecnologia e maior dependência de condições climáticas favoráveis ^58^.

A perda na produtividade resultante dos eventos climáticos extremos gera declínio nos rendimentos dos agricultores e trabalhadores agrícolas, reduzindo também a disponibilidade de alimentos *in natura*, cujo reflexo se vê no menor abastecimento de alimentos nos mercados locais. Em regiões rurais remotas, além do baixo nível de infraestrutura e saneamento, o acesso à alimentação saudável também é comprometido, afetando a quantidade e a qualidade dos alimentos consumidos nas propriedades rurais ^4^ ^,^ ^42^ ^,^ ^58^.

Esse contexto pode resultar ainda no aumento do desemprego, culminando no deslocamento e migração das populações rurais, que passam a se concentrar em áreas urbanas marginalizadas, elevando sua exposição aos eventos climáticos extremos ^4^ ^,^ ^42^ ^,^ ^54^. A maior urbanização contribui para o aumento dos extremos de temperaturas e dificulta o escoamento das chuvas ^1^. Ademais, o êxodo dos pequenos produtores favorece a ampliação da área de plantio para os grandes produtores - que emitem mais GEE e exigem elevada quantidade de água ^4^.

Essa realidade vem sendo vivenciada no Brasil, que observa sua população urbana atingir sua maior proporção (87,4%) nos últimos 22 anos, enquanto a população rural reduziu em 33,8%, percentual que representa cerca de 25,6 milhões de pessoas (12,6% da população) ^59^.

Logo, pode-se considerar que o sistema alimentar hegemônico retroalimenta as desigualdades e estas, por sua vez, contribuem sobremaneira para que o sistema alimentar continue cada vez mais injusto, na medida em que atinge de forma mais expressiva populações específicas (Figura 1, via (d.3), conexão de vulnerabilidade).

Na área rural, as mulheres, responsabilizadas primariamente por garantir a água e os alimentos dentro do domicílio, sofrem desproporcionalmente o efeito das secas causadas - ou agravadas - pelas mudanças climáticas ^4^. A maior distância para coletar a água aumenta o risco às diversas formas de violência baseada no gênero (incluindo violência sexual, sexo transacional, gravidez não intencional e casamento infantil, entre outros), realidade que tem sido vivenciada por meninas e mulheres no semiárido brasileiro (Figura 1, via (d.4), conexão direta) ^60^. Mulheres grávidas, assim como as lactantes e as crianças, são ainda mais sensíveis aos riscos relacionados aos eventos climáticos extremos devido à maior vulnerabilidade às morbidades, como doenças infecciosas, e às necessidades nutricionais aumentadas ^61^.

### O impacto das mudanças climáticas e da insegurança alimentar na má nutrição infantil

As mudanças climáticas impactam a saúde e o estado nutricional das crianças menores de cinco anos de forma direta ou indireta. A forma direta se refere às temperaturas elevadas que aumentam o risco de aborto; e a maior exposição aos poluentes atmosféricos e alérgenos que eleva os riscos de infecção respiratória e asma na infância, nascimento prematuro, baixo peso ao nascer e atraso do desenvolvimento infantil ^62^ ^,^ ^63^ ^,^ ^64^ ^,^ ^65^. A forma indireta pode ser mediada pela insegurança alimentar e se manifesta de formas diversas. De modo geral, a insegurança alimentar grave e a fome estão associadas à desnutrição, enquanto a insegurança alimentar leve e moderada estão, paradoxalmente, associadas à obesidade ^2^. 

Como mencionado, a inter-relação entre as pandemias de desnutrição, da obesidade e das mudanças climáticas é estabelecida no modelo da sindemia global. Essas pandemias interagem entre si e compartilham determinantes sociais comuns, entre os quais o sistema alimentar hegemônico e a concentração de poder e riqueza entre uma minoria de corporações ^2^. Essas características se conectam ao modelo discutido neste ensaio ao abordarem as desigualdades sociais e o sistema alimentar hegemônico como determinantes da má nutrição infantil. 

Essa compreensão também se alinha à estrutura conceitual proposta pelo Fundo das Nações Unidas para a Infância (UNICEF) ^23^, que hierarquiza os determinantes da nutrição materno-infantil em três níveis: o nível amplo, os determinantes facilitadores se referem às condições políticas, recursos ambientais, sociais e econômicos e se conectam à dimensão das desigualdades sociais do modelo aqui discutido; os determinantes subjacentes, ligados aos serviços e práticas de alimentação e nutrição nas casas, comunidades e ambientes alimentares, refletindo as dimensões da insegurança alimentar, insegurança hídrica e sistemas alimentares; e os determinantes imediatos, que envolvem a alimentação saudável e os serviços e práticas adequados para a boa nutrição, relacionados à dimensão da má nutrição infantil, os quais podem prevenir suas manifestações ^23^.

Evidências dessas inter-relações vêm sendo publicadas nos últimos anos ^34^ ^,^ ^66^ ^,^ ^67^. Períodos prolongados de seca são associados ao impacto na produção e no acesso aos alimentos por mulheres indígenas da zona rural da África, levando a nascimentos prematuros ^66^. Por sua vez, os extremos de secas ou inundações foram associados ao menor crescimento linear e ganho de peso insuficiente em crianças menores de cinco anos no Nepal e Uganda, devido à redução na produção de alimentos ^34^. Assim, a desnutrição fetal e infantil resultante desse contexto tende a elevar os riscos de obesidade na infância tardia e vida adulta ^67^ (Figura 1, via (e.1), conexão direta).

Crianças com excesso de peso, por sua vez, são mais suscetíveis às doenças relacionadas ao calor e o clima desconfortável pode levar a uma redução ainda maior da atividade física ^68^ ^,^ ^69^. Além disso, o aumento da frequência dos eventos climáticos extremos reduz a produção de frutas e hortaliças, elevando seus preços, enquanto os ultraprocessados seguem amplamente disponíveis, pois dependem de cadeias agrícolas mais estruturadas e menos vulneráveis a esses eventos ^2^. Consequentemente, os seus preços tendem a ser menores, havendo preferência pelo seu consumo, principalmente pelas famílias com menor renda e em condição de insegurança alimentar ^2^ ^,^ ^37^. Ademais, estima-se que a crescente proporção da população com excesso de peso tende a elevar o consumo de alimentos e bebidas devido a maiores demandas metabólicas, sendo, portanto, necessário o aumento da produção agrícola e, consequentemente, das emissões de GEE ^69^. 

Esse cenário também vem crescendo no Brasil. Entre 2006 e 2019, houve aumento de quatro pontos percentuais na prevalência de excesso de peso entre as crianças menores de cinco anos (6% *vs*. 10,1%) ^70^. Cerca de 80% dessas crianças consomem, com frequência, alimentos ultraprocessados, como biscoitos doces ou salgados e bebidas açucaradas ^71^. Entre a população com baixa renda, esse padrão pode ser um reflexo da falta de acesso físico e financeiro aos alimentos de qualidade ^70^ ^,^ ^72^. 

Em contrapartida, a obesidade pode ser prevenida por práticas alimentares saudáveis nos primeiros anos de vida, como o aleitamento humano e a alimentação complementar ^73^. Os eventos climáticos extremos, no entanto, podem precipitar a interrupção do aleitamento humano devido à falta de suporte e de ambientes privados e seguros para amamentar, e o desconhecimento de estratégias para sua manutenção por mães ou cuidadores ^74^ ^,^ ^75^. Em 2019, a prevalência de amamentação continuada até um ano foi 52,1% no Brasil ^76^, valor aquém das recomendações ^73^. As fórmulas infantis, por sua vez, geram resíduos e possuem risco de menor disponibilidade na ocorrência dos eventos climáticos extremos, além de representar mais uma fonte potencial de água contaminada ^62^ ^,^ ^75^. 

O baixo acesso à água potável e segura (captada pelo indicador de insegurança hídrica) também se associa ao maior risco de desnutrição, na medida em que a água é utilizada para higienização dos alimentos e preparo de refeições. Cabe também considerar que a contaminação da água favorece a incidência de doenças diarreicas, que comprometem o crescimento e o desenvolvimento infantil, além de representarem uma das principais causas de mortalidade nessa faixa etária ^77^ (Figura 1, via (e.3), conexão direta). 

Fenômenos climáticos, como El Niño e La Niña, e temperaturas mais altas estão associados à redução da diversidade alimentar - um dos indicadores da alimentação complementar adequada ^78^ - de crianças com idade entre 9 e 36 meses, comprometendo a qualidade e quantidade da sua alimentação ^33^. No Brasil, as crianças de 6 a 11 meses representaram o grupo que menos consome frutas e hortaliças (25% de não consumo) e cuja alimentação teve menor diversidade alimentar mínima (46,8%) ^71^. 

A baixa diversidade alimentar está relacionada ao baixo consumo de micronutrientes, aumentando o risco de deficiências e a vulnerabilidade a doenças infecciosas transmitidas por água ou alimentos. Crianças com anemia são mais suscetíveis à infecção por malária, cuja prevalência cresce com as mudanças climáticas, porém, a suplementação de ferro utilizada no tratamento pode agravar a infecção ^79^. A carência de vitamina A agrava danos à mucosa intestinal e eleva a mortalidade por diarreia, enquanto a deficiência de vitamina D está associada a maior risco de obesidade e comprometimento do crescimento infantil ^80^ ^,^ ^81^. 

Em 2019, entre as crianças brasileiras de 6 a 59 meses, as deficiências de vitamina B12 (14,2%) e de zinco (17,8%) foram mais prevalentes, enquanto outros nutrientes apresentaram prevalências mais baixas de suas deficiências, como a anemia ferropriva (3,5%), a hipovitaminose A (6%) e a insuficiência de vitamina D (4,3%) ^82^. Nessa mesma população, 47,1% das crianças conviviam em famílias com algum grau de insegurança alimentar, sendo 3,8% delas em nível grave ^83^, evidenciando a relação entre insegurança alimentar e má nutrição na infância ^6^.

Assim, os eventos climáticos extremos podem aumentar ou agravar a má nutrição entre as crianças menores, o que pode torná-las mais vulneráveis às mudanças climáticas; enquanto a má nutrição pré-existente pode elevar a vulnerabilidade da saúde e estado nutricional dessas crianças aos eventos climáticos extremos (Figura 1, via (e.2), conexão de vulnerabilidade).

Como reflexo das desigualdades, as manifestações da má nutrição entre as crianças brasileiras se concentram mais em algumas regiões e populações específicas, como a região Norte, e entre as crianças de cor/raça preta ou parda ^13^ ^,^ ^71^ ^,^ ^82^. Infere-se, portanto, que as crianças que residem em domicílios mais pobres, principalmente meninas, negros, indígenas, quilombolas, ou pertencentes a outros povos e comunidades tradicionais, ou grupos excluídos socialmente, têm o risco à má nutrição aumentado se vivem no contexto das mudanças climáticas (Figura 1, via (e.4), conexão direta).

Esse cenário pode gerar pressão adicional sobre os sistemas de saúde e de proteção social, impactando ainda mais os direitos da criança de acesso adequado à alimentação e saúde.

## Considerações finais

O modelo conceitual proposto neste ensaio contribui para a compreensão das interações entre as mudanças climáticas, a insegurança alimentar, a saúde e a nutrição infantil e como estas são agravadas pela insegurança hídrica e as desigualdades sociais. Destaca ainda o papel do sistema alimentar hegemônico vigente no Brasil, que intensifica tanto as mudanças climáticas quanto a insegurança alimentar e as desigualdades. Além disso, contribui para elucidar lacunas nos estudos sobre mudanças climáticas ao abordar a insegurança alimentar sob a perspectiva do acesso aos alimentos, medido pela EBIA, e ao reconhecer as desigualdades sociais como determinantes centrais dessas relações. 

O modelo conceitual, assim, aponta os caminhos de vulnerabilidade e oportunidades de intervenção, além de orientar futuras pesquisas, no sentido de assegurar saúde e nutrição adequadas para as crianças brasileiras no contexto das mudanças climáticas. Pode favorecer, ainda, a formulação, a implementação e o monitoramento de políticas públicas com ações articuladas e multissetoriais de adaptação e mitigação às mudanças climáticas no Brasil.

## Data Availability

Não se aplica.
